# Exploring the influence of Chinese online patient trust on telemedicine behavior: insights into perceived risk and behavior intention

**DOI:** 10.3389/fpubh.2024.1415889

**Published:** 2024-08-23

**Authors:** Chang Liu, Jiaqi Wang, Ran Chen, Wusi Zhou

**Affiliations:** School of Public Health Management, Hangzhou Normal University, Hangzhou, China

**Keywords:** telemedicine, online patient trust, perceived risk, online consumer behavior, technology acceptance mode/theory of planned behavior

## Abstract

As a supplement to medical services, telemedicine is of great significance to alleviate the shortage of health resources in China. Based on the traditional consumer behavior measurement model the Technology Acceptance Mode/Theory of Planned Behavior (TAM/TPB), this paper divides online patient trust into six dimensions: perceived risk, personal trust tendency, doctors’ credibility, hospitals’ credibility, websites’ credibility, and system guarantee. On this basis, a structural equation model (SEM) was used to explore the influence of each dimension of online patient trust on online patient intention, behavior choice, and pre-factors. A total of 582 valid questionnaires were distributed to selected patients with experience in using mobile healthcare services in the vicinity of hospitals and communities, as well as to users who shared their experiences in the discussion forums of mobile healthcare websites. The results show that online patient trust has a significant positive impact on telemedicine behavior intention selection, with a standardized path coefficient being as high as 0.866. Doctors’ credibility, system guarantee, and website credibility have significant positive effects on online patient trust, with standardized path coefficients of 0.401, 0.260, and 0.226, respectively. Hospital trustworthiness and personal trust propensity have no significant effect on online patient trust. Perceived risk has a significant negative effect on online patient trust, with a standardized path coefficient of −0.118. The research findings suggest that health departments and mobile healthcare providers can enhance mobile healthcare services by considering the patients’ perspectives, elevate their online trust levels, and foster a deeper understanding, safety consciousness, and confidence in telehealth services. On this basis, it can be concluded that only the participation of government, medical subjects, and online patients can effectively reduce perceived risks, improve perceived characteristics of online patients, enhance online patient trust, and promote the real willingness and behavior choice for online medical services, effectively improving the positive role of telemedicine in increasing health benefits to people.

## Introduction

In recent years, the aging of the population and chronic diseases have brought great new challenges to the field of health care in China, even as overall medical resources remain scarce and uneven distribution still exists. Issues also exist for service efficiency and accessibility. The nearly one-fifth of the world’s population that live in China are limited to less than 2% of global medical resources, and high-quality medical resources are mostly concentrated in the more developed Eastern and urban areas (1). The Chinese government and society have been closely monitoring the development of telemedicine and exploring effective solutions to address the issue of uneven distribution of high-quality medical resources by utilizing new technological means. Since the 1990s, the rapid development of telemedicine in China has been promoted by continuous growth in the demand for telemedicine services. However, at present, telemedicine in China is not fully reflected in law and administrative systems and regulated at the national level; moreover, management is insufficient, some doctors have a one-sided understanding of telemedicine, and both doctors and patients have insufficient understanding of telemedicine. This situation makes it difficult to effectively address the demand for health services from a population experiencing aging and increased chronic diseases but also increased birth rates. Therefore, this study sets out to explore the impact of online patient trust on medical service use, in order to develop ways to promote online patient trust and telemedicine behavior intentions to meet the growing need of Chinese residents.

With the development of network communication technology and computer technology in China, the affordances of telemedicine have been continuously enriched, its advantages have become increasingly prominent, and it has ushered in major development opportunities. According to the World Health Organization, 2020–2030 may be the decade in which digital technology reshapes the health care system, and the telemedicine industry is accelerating. According to a survey by the China Internet Network Information Center (CNNIC), as of December 2020, the number of netizens in China has reached 989 million; among them, 99.7% use mobile phones to access the Internet; and 215 million are online medical users, or 21.7% of the total (1). However, these netizens will also face various risks when using telemedicine, such as perceived risk, credit crisis, institutional risk, and so on. This means it is urgent to cultivate awareness, security awareness, and confidence in telemedicine services and promote the development of a diversified telemedicine industry. Network risk aggravates the negative perception of medical consumers and reduces people’s trust in telemedicine. The research shows that trust increases with income and education (2); as an informal system, trust is recognized as an important social capital and is influenced by regional economic growth and social progress (3). In an environment of greater trust, people are more abiding by the values of honesty and trustworthiness, which in turn reduces risk (1). Some scholars have discussed this issue in the context of e-commerce, where risk mostly refers to the uncertainty of asset security and privacy exposure. Empirical research in that context shows that psychological risk has no significant impact on users’ willingness to use (4). It is worth considering that the vigorous development of mass consumption online platforms and the enthusiastic participation of ordinary users who lack purchasing experience and background knowledge contradict the existing research conclusions. At the same time, perceived network insecurity and reduced consumer participation do not fully support the relevant research conclusions (5). Some sellers or suppliers do not fully assess risks for demanders, and tend to ignore the impact of psychological and emotional risks (6, 7).

In addition, the model of technology acceptance (TAM) and the theory of planned behavior (TPB) effectively explain consumer adoption behavior. In 1989, Davis applied rational behavior theory to propose a model for studying users’ acceptance of information systems, which became known as the Technology Acceptance Model (TAM). TAM seeks to explain the adoption of new technologies by individuals, identifying perceived ease of use and perceived usefulness as key determinants of IT adoption. Building on this foundation, TPB incorporates subjective norms and perceived behavioral control, offering a more comprehensive explanation and prediction of individuals’ behavioral strategy choices. But some behavior models only explore the independent influence of trust or risk on behavior, and only confirm that the brand trust of a product or service has a significant positive effect on its extended use, and perceived risk factors are not considered. Only some behavior models discuss the indirect effect of trust on behavior through perceived risk (8).

Up to now, the research on trust degree in telemedicine is still a blank. There is no systematic research on how main factors such as institutional security, hospital, doctor, and website affect individual trust tendency and perceived risk in telemedicine online patients and determine their behavior intention.

Based on an integration model of TAM and TPB, this paper selects online patients as the research object, discusses the hierarchical relationship between the prepositional factors in TAM/TPB and the perception of risk and trust after subdividing the special risk dimension, further establishes the medical service behavior intention model affecting online patients, provides a theoretical basis for rapid and sustainable development of telemedicine in the future, and provides a theoretical basis and practical guidance for medical institutions, governments, and third-party service platforms to understand online patients’ risk perception, consumption confidence, and risk avoidance measures. To sum up, this paper defines the concept of trust in telemedicine, considers that trust is born of risk and exists together with it, and validates this model in the following hypotheses. The remaining parts of this paper are arranged as follows: Next, based on the review of the relevant literature, we put forward the theoretical model and research assumptions. Then, we introduce the research methods, including questionnaire design, sample data collection, and model testing. Finally, we draw conclusions and make policy recommendations.

## Theoretical model

**Figure 1:**
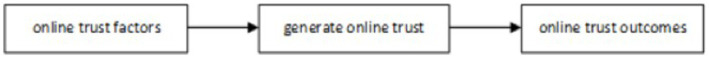
Online trust research framework.

In the absence of risk protection for online platforms, in order to ensure the desired effect, trust plays a good substitute role (9). Online trust is defined as an expected judgment and psychological state in which people believe that some other person will not act against them in an online environment (10). In the new business form of telemedicine, this paper holds that existing general online trust research results or those from different realms are applicable to online patient trust research. We simplified the Shankar Online Trust Model to create a conceptual model consisting of three parts: antecedents of trust, online trust (itself), and trust outcomes, as shown in Figure 1 (11). The online trust model mainly analyzes the relationship between online trust factors, online trust performance, and online trust behavior. The online trust model primarily examines the factors that engender online trust, the factors influencing online trust’s manifestations, and the outcomes of online trust, as well as their interrelationships.

The configuration of various variables primarily relies on the online trust model and perceived risk theory, aligned with the unique characteristics of mobile medical services. The determinants of patients’ online trust are categorized into four dimensions: subjective factors, objective factors, third-party factors, and additional factors. Subjective factors encompass the patient’s individual trust propensity; Objective factors include the credibility of websites, hospitals, and doctors; Third-party factors refer to the relevant regulatory frameworks for mobile healthcare services; Additional factors are the perceived risks by patients. Online trust factors in Figure 1 are usually divided into factors for consumers, websites, businesses, and third parties. A trust relationship is essentially mutual. Generally, the research on trust subjects (trusters) in telemedicine involves patients and medical service providers. Most research on the definition of physician–patient trust is also done from a patient’s perspective, and online patient trust is generally seen as patients’ confidence in their physician’s ability to care and to put the patient’s interests first (12). As for trust objects [(non-)trusted entities], online patient trust can be divided into patients’ trust in medical institutions and trust between patients and doctors (13). Online patient trust can have an impact on health outcomes, etc. (14), and has important implications for the harmony of patients’ health behaviors. This paper adopts the Davis Technology Acceptance Model (TAM) (15), and focuses on the joint effect of three main influencing factors—perceived risk, personal trust tendency, and medical issue—on online patient trust and behavior. Ajzen’s Theory of Planned Behavior (TPB) (16) emphasizes that behavioral intentions are determined by variables other than the perceived behavioral attitudes of online patients. Focusing on these topics, scholars have shown that several models have some explanatory power when applied separately, but there are obvious deficiencies in each model, and the integrated interpretation significantly improves explanatory power (17).

TAM and TPB models have been widely used to study the purchasing behavior of consumers in various fields. As online consumers become familiar with the business environment of online platforms, the assumption that perceived risk affects trust has been put forward (18). Based on the uncertainty of internet transactions and the importance of consumers’ psychological risk expectations, more and more scholars have explored the impact of pre-factors on trust with perceived risk as an intermediary, to comprehensively consider the relationship between risk, trust, and consumer behavior. Some scholars have expanded their research in the field of e-commerce, applying TAM models widely to Internet service platforms and using PLS methods to demonstrate that consumer perception significantly affects behavioral intention to receive online services (19). Others have incorporated TAM, perceived risk, and trust into their research framework to explore the relationship among these factors; the results have shown that the reduction of perceived risk can quickly establish consumers’ trust in online services and indirectly affect trust through the intermediary of perceived risk (20). Other researchers have divided perceived risk into several types, such as operational risk, psychological risk, and social risk, to study the impact of trust (21).

**Figure 2:**
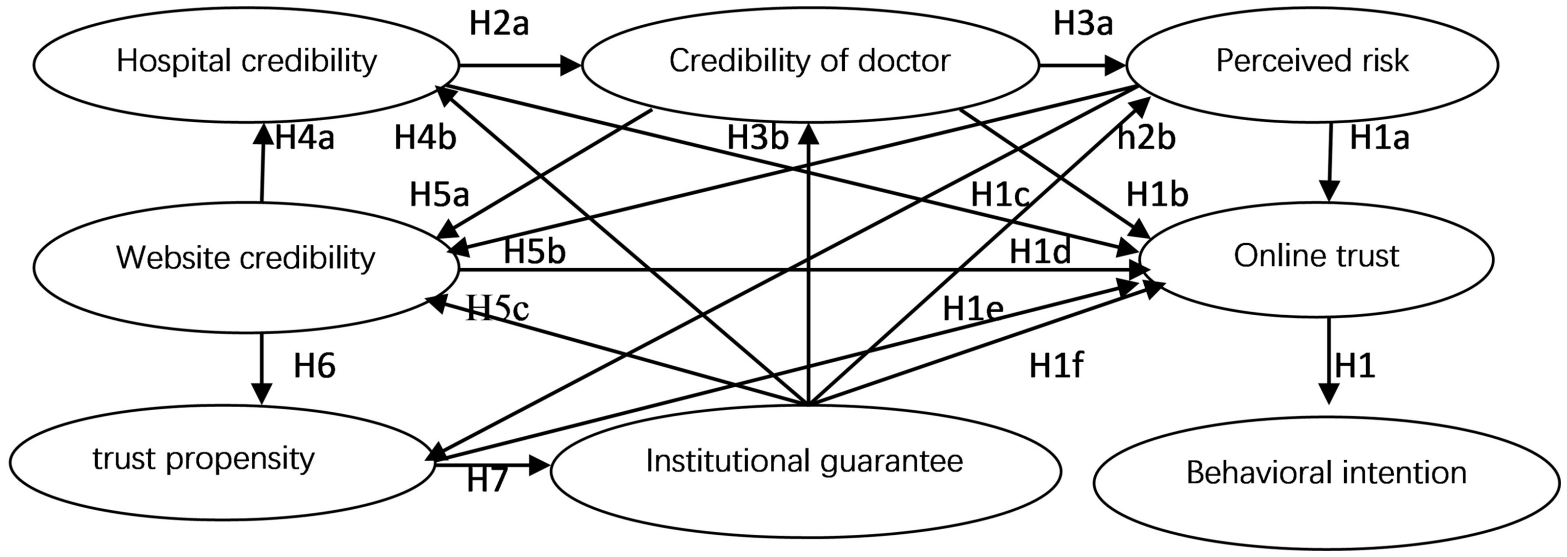
Research model.

To sum up, first, there are more effective in reducing online patient risk perception and enhancing online patient willingness. Based on this logic, perceived risk, personal trust tendency, online medical service providers, and policy environment are selected as the key pre-variables that affect online patient trust intention. Moreover, we analyze the influence of different dimensions of risk on online patient behavior in telemedicine services. Second, although the TAM and TPB models have been revised step by step in the existing literature, there has been no careful examination of whether there is any progressive logical relationship between the theories. Third, perceived risk only appears in a single dimension in many literatures, but this research posits that perceived risk is a multidimensional concept and should be refined according to the special attributes of the research objects. Therefore, from the perspective of online consumers, the perceived characteristics of telemedicine are more effective in reducing online patient risk perception and enhancing online patient willingness. Based on this logic, perceived risk, personal trust tendency, online medical service providers, and policy environment are selected as the key pre-variables that affect online patient trust intention. Moreover, we analyze the influence of different dimensions of risk on trust, and finally elaborate a model of online patient trust, medical service subject behavior, and institutional guarantee model. This study model is shown in Figure 2.

## Research hypotheses

### Online patient trust and behavioral intention

From a sociological point of view, trust is usually interpreted as an intention to act or as an act *per se* that reflects one party’s belief that the other party’s behavior is trustworthy or will not result in loss. Trust is an important factor in building long-term, stable trading relationships and has a significant positive impact on individual consumer behavior (18, 22). There is a positive correlation between trust and behavioral intention from the perspective of e-commerce, which has not been verified in telemedicine studies; with this in mind the following hypothesis is proposed:

*H*1: Online patient trust has a positive effect on medical behavior intention.

### Risk perception and online patient trust

As one of the basic characteristics of trust, online patients’ perceived risk usually affects their behavior intention, together with trust. Trust reflects whether consumers believe in each other’s internal tendencies, and is based on experience and the formation of a general psychological tendency to rely on others (23). Empirical studies show a negative correlation between perceived risk and online consumer trust (24, 25). At present, scholars in China, where this study is set, and internationally have studied the dimensions of perceived risk by identifying specific types of risk pertaining to different research objects in specific situations. Jacoby and Kaplan divided consumers’ perceived risk into the following: financial risk, functional risk, physical risk, psychosocial risk, and social risk (26). Stone and Gronhaug verified the existence of six risk dimensions: financial, performance, physical, psychological, social and time, with 88.8% explanatory power to overall perceived risk (27). At the same time, the roles of price equity and environmental concerns in the perception of environmental knowledge and ecological awareness between consumer behavior have a certain impact on institutional security (28). Yang et al. clearly defined perceived risk types as economic, functional, security, time, privacy, social, service, and psychological risks (29).

Based on the relevant research, this paper believes that a certain dimension can indeed partially reflect the overall perceived risk, and it is included in the set of perceived risk dimensions. Then, according to telemedicine characteristics and service behavior, a few mismatched types in the dimension set are eliminated, and special risk dimensions are added. Finally, with reference to Lee et al. on the moderating effect of perceived risk on the relationship between fashion consumption motivation and purchase intention (30) and to the dynamic formation mechanism of perceived risk in multi-stage decision-making (31), we will directly ask consumers about their risk perception and determine the five perceived risks suitable for telemedicine: security, privacy, social, psychological, and time. Then, we conduct principal component analysis on the finalized risk set and extract two factors—reliability risk and institutional guarantee—to examine their relationships with trust. Among these, credibility risk reflects online patients’ assessment of potential risk possibility according to their subjective emotions, and it is easy to self-control and adjust the degree of perceived risk. System guarantee risk is different; it occurs generally due to changes in the external environment, resulting in greater financial or other loss risk for the medical treatment main body. In view of this, the following hypotheses are proposed:

*H*1a: Reducing perceived risk effectively increases online patient trust.

*H*1b: Doctor credibility helps increase online patient trust.

*H*1c: Hospital credibility effectively enhances online patient trust.

*H*1d: Website credibility effectively enhances online patient trust.

*H*1e: Personal trust tendencies effectively increase online patient trust.

*H*1f: Institutional guarantees effectively increase online patient trust.

### Perception characteristics and trust based on TAM/TPB theory

Given the huge potential for growth in telemedicine usage, its emergence has provided some convenience and benefits to online patients. However, under the current buyer’s market conditions, in which consumers are in charge, online patients will perceive and value telemedicine based on their own personal trust tendencies, perceived risks, and institutional guarantees, which will profoundly affect online medical trust. According to the definition of perceived characteristics of TAM and TPB schools, perceived risk refers to online patients’ belief that the use of telemedicine can improve their health benefits, and personal trust tendency refers to online patients’ value judgments on the health benefits of new medical services, while the system guarantee reflects the influence of the external environment of telemedicine on the relevant medical service areas. Many studies on e-commerce show that consumer perception characteristics and trust are not independent of one another; perceived risk, personal trust tendency, and institutional guarantees will all impact trust (32). In view of this, the following six hypotheses are proposed:

*H*2a: Doctor credibility significantly affects perceived risk.

*H*2b: Institutional guarantee effectively reduces perceived risk.

*H*3a: Hospital credibility has a positive impact on doctor credibility.

*H*3b: Institutional guarantee effectively increases doctors’ credibility.

*H*4a: Website credibility has a positive impact on hospital credibility.

*H*4b: Institutional guarantee effectively increases the credibility of hospitals.

In previous research, consumers’ perceived characteristics and perceived risk are regarded as the same type of antecedents to study their combined influence on the intention of Internet users. In fact, for innovative Internet services, consumers make subjective assessments on the premise of self-perception, from which they derive perceived risks. Yang et al. responded to this point of view (20), proposing that online patients’ perceived risk and personal trust tendencies would negatively affect the overall perceived risk. So far, however, there is no relevant literature that considers the differential impact of consumer perception characteristics on different risk categories. Based on the above analysis, the following (sub-)hypotheses are put forward:

*H*5a: Doctor credibility has a positive impact on website credibility.

*H*5b: Perceived risk has a positive impact on website credibility.

*H*5c: Institutional guarantee effectively enhances website credibility.

*H*6: Website credibility has a positive effect on personal trust propensity.

*H*7: Personal trust tendency has positive influence on institutional security.

## Methods

### Questionnaire design

Inclusion criteria: (1) Over 18 years old without cognitive impairment; (2) Have experience using telemedicine. The sample size is determined by Kendall’s sample estimation method and is calculated at 10–20 times the total number of items in the questionnaire (33). Taking into account the potential for invalid questionnaires and increasing the sample size appropriately, a total of 600 questionnaires were distributed, with 590 being collected, representing a response rate of 98.3%. After discarding incomplete, haphazardly filled, or incorrectly completed questionnaires, a total of 582 valid questionnaires were received, achieving an effective response rate of 97%. The questionnaire is divided into two parts: The first part, with a total of 8 questions, is mainly on the general demographic characteristics of the respondents, their health status, and the frequency of telemedicine services used, such as: gender, age, education, marital status, occupation, whether suffering from chronic diseases, average daily online hours, and frequency of using mobile medical services. The second part, the Online Patient Trust Scale, has a total of 34 items (Table 1), measured from different aspects: personal trust propensity, website credibility, hospital credibility, doctor credibility, system security, perceived risk, online patient trust, and behavior intention, in all 8 variables. The questionnaire primarily comprises two sections: respondents’ basic information and an online patient trust scale. The scale design incorporates research from relevant scholars, and building on measurement items for specific variables from domestic and international literature, adjustments and modifications were made to align with the mobile healthcare context of this study, thereby ensuring the scale’s content validity. To validate the survey results, we consulted with relevant experts and made revisions prior to the official distribution of the questionnaire. This was followed by a small-sample pre-survey using the revised questionnaire. Data was collected using convenient sampling via both online and offline channels. Online research utilized the professional platform *Wenjuanxing* to distribute and collect questionnaires in the user forums of mainstream mobile medical websites. Offline research involved the team conducting surveys by intercepting residents in the vicinity of hospitals and communities. Prior to questionnaire distribution, respondents were asked whether they had any experience with mobile medical services; those responding affirmatively were then invited to complete the questionnaire on the spot.

**Table 1:** Latent variable structure and source.

Dimension	Measurement items	Source
	Behint1: Willingness to use mobile health services again	
Behavioral intention (Behint)	Behint2: Willing to recommend mobile health services to others	(34)
	Behint3: Makes positive comments about mobile health care	
	OnlinePT1: High-quality telemedicine service delivery	
Online patient trust	OnlinePT2: Telemedicine can relieve stress	(14)
(OnlinePT)	OnlinePT3: Telemedicine is reliable	
	OnlinePT4: Telemedicine can meet the demand for medical treatment	
	PERCR1: Fear of monetary damage from telemedicine	
	PERCR2: Fear of harm from inadequate telemedicine	
Perceived risk	PERCR3: Using telemedicine involves more privacy risks	(35)
(PERCR)	PERCR4: Others do not recommend or support mobile medicine	
	PERCR5: Using telemedicine makes me nervous	
	DCC1: I think the doctor is very good	
	DCC2: I think the doctor is a noble man	
Credibility of doctor	DCC3: The doctor will let me know if anything goes wrong	(36–38)
(DCC)	DCC4: I think the doctor understands my ideas about the illness	
	DCC5: I’m sure the doctor will put my medical needs first	
	SECS1: Medicare payment policy introduced to secure telemedicine transactions	
Institutional guarantee	SECS2: Data security makes medical privacy more secure	(39)
(SECS)	SECS3: Internet diagnosis and treatment standard mobile medical information true and reliable	
	HPC1: The hospital has strong comprehensive strength	
	HPC2: The hospital is well known	
Hospital credibility	HPC3: The hospital is able to provide quality medical care	(37)
(HPC)	HPC4: The hospital is responsive to the needs of its patients	
	HPC5: The hospital has a good reputation in my field of illness	
	WBC1: The site has useful page navigation	
	WBC2: The website has a good reputation	
Website credibility	WBC3: The diagnosis and treatment information on this website is true and reliable	Harris (37)
(WBC)	WBC4: The site has a secure payment system	
	WBC5: The site focuses on patient privacy	
	PCT1: I usually trust people	
Trust propensity (PCT)	PCT2: I usually have faith in human nature	(39, 40)
	PCT3: It is easy for me to trust someone	
	PCT4: I tend to trust a person even though I do not know them well	

### Statistical analysis

Data were coded and entered in the database. The basic information for the valid sample (Table 2) was 303 males, accounting for 52.1%, while 44.8% of the respondents were 18–30 years old and those 51 years old and above accounted for 6.9%; moreover, 38.8% had a bachelor’s degree. Next, 56.9% were married; professions represented included enterprise employees, students, public institution staff, self-employed, farmers, service personnel, retirees, and other professional personnel. The number of respondents without chronic diseases is much larger than that of those with chronic diseases. The average daily Internet time is 1–3 h for 32% of respondents, a plurality, and 64.9% of patients used telemedicine services once every 2 months or more. It is obvious that most of the patients use telemedicine services at the initial stage, which is in line with the current development of network medical services in China.

**Table 2:** Descriptive statistics of the sample (*N* = 582).

Characteristic	Characteristic	Frequency (N)	Composition ratio (%)
Gender	Male	303	52.1
	Female	279	47.9
Age (years)	18–30	261	44.8
	31–40	171	29.4
	41–50	110	18.9
	≥51	40	6.9
Educational degree	High-school or below	168	28.9
	Junior college	150	25.8
	Undergraduate	226	38.8
	Master’s or above	38	6.5
Marital status	Unmarried	229	39.3
	Married	331	56.9
	Divorced	20	3.4
	Widowed	2	0.3
Profession	Office staff	69	11.9
	Self-employed persons	81	13.9
	Enterprise employees	183	31.4
	Farmer	31	5.3
	Service personnel	40	6.9
	Student	106	18.2
	Retirees	15	2.6
	Others	57	9.8
Whether chronic disease	Yes	98	16.8
	No	484	83.2
Use mobile health service frequency	Once or more a week	58	10.0
	Once every half a month	71	12.2
	Once a month	75	12.9
	Once 2 months or more	378	64.9
Purpose of using mobile medicine	Learn about health information	313	53.8
	Hospital registration	182	31.3
	Medical consultation	87	14.9
Average daily online time	Less than 30 min	14	2.4
	30 min to 1 h	61	10.5
	1–3 h	186	32.0
	3–5 h	152	26.1
	More than 5 h	169	29.0

The total trust score is calculated by summing the scores of each item within the online patient trust dimension. The Shapiro–Wilk test reveals that the total trust score of online patients does not follow a normal distribution, as indicated by *p* < 0.05. However, the histogram shows that the total trust score of online patients follows a normal distribution with more scores in the middle and fewer on both sides, with no obvious extreme values. Therefore, a two-sample t-test was used to test for differences in binary variables such as gender and chronic disease status; One-way analysis of variance (ANOVA) was used to test for differences in multiple categorical variables such as age, education level, marital status, occupation, average daily internet usage, and frequency of mobile healthcare service usage. There is no significant difference in online patient trust based on gender, education level, occupation, presence of chronic diseases, and frequency of mobile healthcare service usage; Significant differences are observed in age, marital status, and average daily internet usage. Pairwise comparisons reveal that patients of different ages have different online trust scores (*p* < 0.05). Patients aged 31–40 have the highest online trust scores, followed by patients aged 41–50 and 18–30, while patients aged 51 and above have the lowest scores; The online trust scores of patients vary depending on their marital status (*p* < 0.05), with divorced patients scoring the highest; The difference in online trust scores among patients with different average daily internet usage hours was statistically significant (*p* < 0.05).

Thus, users tended to be relatively healthy and educated younger men working across a range of professions, with moderate internet and telemedicine use.

## Research results and analysis

### Descriptive statistics and correlation analysis

The TPB theoretical model primarily targets an individual’s behavioral intentions, comprising three variables: attitude, subjective norm, and perceived behavioral control. The TAM theoretical model primarily targets users’ acceptance of information technology, consisting of two variables: perceived usefulness and perceived ease of use. In the practical application of telemedicine, the TPB model is apt for explaining and predicting the behavioral intentions of online patients, whereas the TAM model is apt for explaining and predicting users’ acceptance of online medical information technology. Additionally, the TPB model also takes into account the influence of social pressure on individual behavioral intentions, thereby offering distinct advantages in explaining and predicting social behaviors like institutional safeguards. Trust propensity, perceived risk, institutional guarantee, credibility of doctor, hospital credibility, website credibility, and behavioral intention had significant correlations with online patient trust, indicating that further testing with the SEM is suitable (Table 3).

**Table 3:** Correlation coefficient of each variable in telemedicine trust dimension (*N* = 582).

	1	2	3	4	5	6	7	8
1. Institutional guarantee	1							
2. Trust propensity	0.522^***^	1						
3. Website credibility	0.647^***^	0.508^***^	1					
4. Perceived risk	−0.201^***^	−0.098^***^	−0.105^***^	1				
5. Hospital credibility	0.733^***^	0.452^***^	0.711^***^	−0.147^***^	1			
6. Credibility of doctor	0.750^***^	0.458^***^	0.711^***^	−0.163^***^	0.793^***^	1		
7. Online patient trust	0.747^***^	0.446^***^	0.708^***^	−0.263^***^	0.713^***^	0.795^***^	1	
8. Behavioral intention	0.647^***^	0.386^***^	0.612^***^	−0.228^***^	0.617^***^	0.688^***^	0.866^***^	1

*** *p* < 0.001, two-tailed test.

### Reliability and validity test of the measurement model

First, SPSS22 was used to test the suitability of the eight dimensions of perceived risk by reducing the dimension of the dataset according to the principal components. The Kaiser–Meyer–Olkin (KMO) test value was 0.936 and the statistical value of Bartlett’s sphere test value was 0.000, which indicates that principal component analysis is feasible (39, 38, 41). Chi-square is 1038.956, and probability is 0.000.

Second, principal component analysis was performed and the following results were obtained (Tables 4, 5). The cumulative variance contribution rate of the six principal components extracted in Table 4 is 67.839%, which shows that the method has sufficient explanatory power. The first principal component in Table 3 is summarized as hospital credibility; the detailed factors are hospital visibility, service quality, patient response to demand, and hospital reputation. The second principal component is online patient trust, and the detailed factors are the quality of telemedicine services, reliability, and satisfaction of medical needs. The third component is website credibility. The fourth principal component is individual trust tendency. The fifth and sixth principal components are summarized as perceived risk, with detailed factors belonging to different measurement dimensions (Table 5).

**Table 4:** Total variance explained.

Component	Initial feature			Load sum of squares			Rotate square and load		
	Total	Variance (%)	Cumulative%	Total	Variance (%)	Cumulative%	Total	Variance (%)	Cumulative%
1	11.604	37.319	37.319	11.604	37.319	37.319	5.300	17.046	17.046
2	3.736	12.016	49.335	3.736	12.016	49.335	4.623	14.866	31.912
3	1.719	5.528	54.863	1.719	5.528	54.863	3.148	10.124	42.036
4	1.457	4.687	59.550	1.457	4.687	59.550	2.574	8.278	50.315
5	1.211	3.895	63.446	1.211	3.895	63.446	3.082	9.910	60.225
6	1.114	3.581	67.027	1.114	3.581	67.027	2.115	6.802	67.027

Extraction method, principal component analysis.

**Table 5:** Rotating principal component matrix.

Indicators	1	Indicators	2	Indicators	3	Indicators	4	Indicators	5	Indicators	6
HPC4	0.784	OnlinePT4	0.731	WBC3	0.762	PCT3	0.781	PERCR5	0.893	PERCR1	0.774
HPC5	0.780	OnlinePT3	0.731	WBC4	0.715	PCT4	0.762	PERCR4	0.844	PERCR3	0.733
HPC2	0.748	OnlinePT1	0.714	WBC5	0.710	PCT2	0.702	PERCR2	0.646		
HPC3	0.742	Behint2	0.705	WBC1	0.708	PCT1	0.696				
DCC1	0.706										

Extraction method: principal component analysis, 1, 2, 3, 4, 5, and 6 represent principal components respectively; Rotation method: Kaiser standardized maximum variance method.

Third, based on the criterion that Cronbach’s α coefficient is greater than 0.7, widely used in academia (42–44), and then according to Churchill’s criterion that the overall correlation coefficient of that should not be less than 0.5 (45), the reliability of the model is tested. The results are shown in Table 4. The 8 dimensions of the model are behavioral intention, online patient trust, perceived risk, doctors’ credibility, institution guarantee, hospital credibility, website credibility, and personal trust tendency. The coefficients of Cronbach’s α were higher than 0.8 and the CITC value was higher than 0.7, indicating that the questionnaire had high reliability.

Finally, confirmatory factor analysis (CFA) is used to analyze the convergence validity of the measurement model. The structural model can be further evaluated only if the measurement model is moderately acceptable. The consolidated data (Table 6), the load of standardized factors in all dimensions, was more than 0.6 (STD.; column data in Table 4) and significant; the combined reliability (C.R. value) was more than 0.7, and the average AVE value was more than 0.5. All the above results met the Hair et al. and Fomell et al. validation criteria (46, 47): ① factor load greater than 0.5; ② combination reliability greater than 0.8; ③ average ANOVA extraction value greater than 0.5. Therefore, the model has good convergence validity. At the same time, SPSS exploratory factor analysis was used in this paper to verify whether a single factor could explain most of the variation. The calculated result was that the cumulative variance explained rate was 40.287%, which was less than the standard value of 50% without common method deviation.

**Table 6:** Reliability and convergent validity analysis of measurement model.

Component	Measurement index	Estimate	S.E.	*t*	STD.	C.R.	AVE	Cronbach’s α	CITC
Behint	Behint1	1			0.885^***^	0.877	0.705	0.876	0.803
	Behint2	0.959	0.039	24.617	0.809^***^				0.742
	Behint3	0.944	0.038	24.688	0.823^***^				0.739
OnlinePT	OnlinePT1	1			0.819^***^	0.852	0.593	0.856	0.736
	OnlinePT2	0.866	0.052	16.603	0.645^***^				0.616
	OnlinePT3	0.969	0.043	22.495	0.812^***^				0.734
	OnlinePT4	1.013	0.047	21.494	0.791^***^				0.723
PERCR	PERCR1	1			0.725^***^	0.835	0.508	0.831	0.516
	PERCR2	1.564	0.119	13.095	0.878^***^				0.726
	PERCR3	1.115	0.080	13.942	0.651^***^				0.624
	PERCR4	1.220	0.102	11.971	0.672^***^				0.680
	PERCR5	1.132	0.102	11.101	0.606^***^				0.608
DCC	DCC1	1			0.818^***^	0.889	0.616	0.892	0.739
	DCC2	1.063	0.045	23.684	0.839^***^				0.773
	DCC3	1.026	0.052	19.783	0.745^***^				0.693
	DCC4	0.923	0.044	20.912	0.786^***^				0.768
	DCC5	1.132	0.102	11.101	0.730^***^				0.717
SECS	SECS1	0.950	0.039	25.288	0.859^***^	0.883	0.716	0.884	0.778
	SECS2	1.008	0.042	24.020	0.824^***^				0.764
	SECS3	1			0.855^***^				0.782
HPC	HPC1	0.946	0.040	23.406	0.857^***^	0.926	0.713	0.924	0.782
	HPC2	0.923	0.040	23.289	0.809^***^				0.786
	HPC3	0.944	0.037	25.362	0.859^***^				0.819
	HPC4	0.973	0.036	27.344	0.830^***^				0.822
	HPC5	1			0.866^***^				0.804
WBC	WBC1	0.980	0.060	16.255	0.743^***^	0.870	0.572	0.871	0.665
	WBC2	0.938	0.057	16.329	0.741^***^				0.673
	WBC3	1.083	0.058	18.553	0.822^***^				0.764
	WBC4	0.951	0.050	19.113	0.755^***^				0.716
	WBC5	1			0.716^**^				0.674
PCT	PCT1	0.825	0.067	12.376	0.628^***^	0.785	0.478	0.783	0.551
	PCT2	0.947	0.071	13.333	0.703^***^				0.591
	PCT3	1.050	0.073	14.332	0.757^***^				0.644
	PCT4	1			0.671^***^				0.572

***Indicates significant correlation at the level of 0.01.

### Structural equation model analysis

#### Structural equation model overall fit moderate evaluation

Table 7 details the main fit indicators obtained from structural model testing. Compared with the recommended values given by the adaptation index, except GFI and NFI, which are very close to the reference index values, the fitting values of other adaptation indexes are all within the range of the reference index value.

**Table 7:** Matching index values of structural equation model.

Fitting index	Fitting standard	Modified model
χ^2^	The smaller the better	1038.956
χ^2^/df	<3	2.082
GFI	>0.9	0.905
AGFI	>0.8	0.887
NFI	>0.9	0.922
IFI	>0.9	0.958
CFI	>0.9	0.958
RMSEA	<0.08	0.043

#### Test of research hypothesis

Based on the structural relationship between the latent variables and the estimated values of the normalized path coefficients, COV., and the hypothesis test results (Table 8), it can be seen that all the hypotheses have passed the significance test, and the path coefficients are significant at the confidence level α = 0.001. Actual model and path coefficients (Figure 2) are shown below.

**Table 8:** Hypothesis test results.

Hypothesis	Correlation	Correlation coefficient	COV. value	Conclusion
H1: Online patient trust has a positive effect on medical behavior intention	OnlinePT→ Behint	0.925^***^	0.484	Suitable
H1a: Reduce perceived risk and increase online patient	PERCR→OnlinePT	−0.135^***^	−0.120	Suitable
H1b: Doctor Credibility Increases Online Patient Trust	DCC → OnlinePT	0.386^***^	0.433	Suitable
H1c: Hospital Credibility Increases Online Patient Trust	HPC → OnlinePT	0.031	0.400	–
H1d: Website credibility increases online patient trust	WBC → OnlinePT	0.219^***^	0.381	Suitable
H1e: Personal trust propensity effectively increases online patient trust	PCT → OnlinePT	−0.015	0.222	–
H1f: Institutional Guarantee Effectively Increases Online Patient Trust	SECS→OnlinePT	0.257^***^	0.395	Suitable
H2a: Doctors’ Credibility Significantly Affects Perceived Risk	DCC → PERCR	−0.032	−0.077	–
H2b: Institutional Guarantee to Effectively Reduce Perceived Risks	SECS→PERCR	−0.153	−0.093	–
H3a: Hospital credibility has a positive effect on doctor credibility	HPC → DCC	0.436^***^	0.463	Suitable
H3b: Institutional Guarantee Increases Doctors’ Credibility	SECS→DCC	0.415^***^	0.413	Suitable
H4a: Website credibility has a positive effect on hospital credibility	WBC → HPC	0.309^***^	0.411	Suitable
H4b: Institutional Guarantee Increases Hospital Credibility Effectively	SECS→HPC	0.565^***^	0.416	Suitable
H5a: Positive Impact of Doctor Credibility on Website Credibility	DCC → WBC	0.364^***^	0.398	Suitable
H5b: Perceived risk has a positive effect on website credibility	PERCR→WBC	0.040	−0.049	–
H5c: System Guarantee Increases Website Credibility Effectively	SECS→WBC	0.316^***^	0.352	Suitable
H6: Website credibility has a positive effect on personal trust propensity	WBC → PCT	0.309^***^	0.260	Suitable
H7: Trust Tendency Has Positive Influence on Institutional Security	PCT → SECS	0.392^***^	0.263	Suitable

*** Indicates significant correlation at the level of 0.01; COV represents a covariate that contains all variables.

#### Analysis of perceived risk intermediary effect

In this paper, the Process Intermediate Test is applied to analyze and re-estimate the standard error and the trust interval of indirect effects using the Bootstrap technique, and the Prodclin2 indirect effects trust interval calculation procedure of MacKinnon is used to verify the mediation effect, get data validation results (Table 9). According to Bias-Corrected and Percentile results, the upper and lower intervals do not contain 0, and Z > 1.96 or Z = 1.96, that is, the criteria for determining the existence of indirect effects indicate the existence of total indirect effects of perceived risks; the data of MacKinnon Prodclin2 show that both online patient trust and personal trust have significant indirect effects on perceived risk.

**Table 9:** Total indirect effect report.

Variable	Point-estimate	Multiplying coefficients		Bootstrapping				MacKinnon Prodclin2	
				Bias-corrected		Percentile			
		SE	Z	Lower	Upper	Lower	Upper	Lower	Upper
PCT → OnlinePT	0.331	0.021	−0.898	0.113	0.419	0.115	0.420		
PERC→OnlinePT	0.490	0.076	0.898	−0.182	−0.028	−0.181	−0.028		
SECS→OnlinePT	0.707	0.012	0.113	0.687	0.861	0.683	0.859		
DCC → OnlinePT	0.149	0.059	0.356	0.122	0.707	0.387	0.707		
HPC → OnlinePT	0.642	0.081	0.685	0.387	0.466	0.114	0.459		
WBC → OnlinePT	0.490	0.179	0.753	0.268	0.525	0.268	0.524		
SECS→WBC → OnlinePT								0.181	0.253
DCC → WBC → OnlinePT								0.075	0.183
SECS→WBC → OnlinePT								0.007	0.039

Non standardized coefficient is used for point estimation.

## Discussion

### Effects of telemedicine and perceived risk on online patient trust

First, online patient trust is the main factor affecting willingness to accept service; the path coefficient is as high as 0.925. This shows that when online patients finally make behavioral decisions, they are well judged by their own perception characteristics, and their trust in telemedicine is much higher than their awareness of online risk prevention. Online patients are more inclined to trust innovative things; especially, young people are more curious, and this can easily lead to telemedicine services. Doctors’ credibility, institutional guarantee, and website credibility have significant positive effects on online patient trust, and their path coefficients are 0.401, 0.260, and 0.226, respectively.

Second, empirical data show that perceived risk has a significant negative impact on online patient trust, with a path coefficient of −0.135. This shows that telemedicine’s service level and technical level are not mature, the behavior of online medical treatment tends to be more prudent, and rational, and the trust of online transactions is insufficient. This is consistent with the fact that trust has been incorporated into consumer behavior models in most related studies in recent years. This paper holds that perceived risk is the key to online patient trust: telemedicine market players need to take effective strategies to reduce risk perception, which will further improve online patient trust.

Finally, the data analysis results show that personal trust tendency and hospital credibility do not have a significant impact on online patients. This may be due to people’s lack of awareness of telemedicine, and fact that the credibility of hospitals mainly comes from offline medical treatment. In addition, each hospital does not engage sufficiently in publicity and promotion of its own image. This provides a theoretical basis for all actors in the medical system to encourage residents to actively participate in the implementation of telemedicine services by reasonable optimization and multi-party development, and to reduce network risks by enhancing the confidence of patients and hospitals in telemedicine, to promote a positive role for telemedicine in improving public health benefits.

### Perceived characteristics of online patients can significantly reduce perceived risk

**Figure 3:**
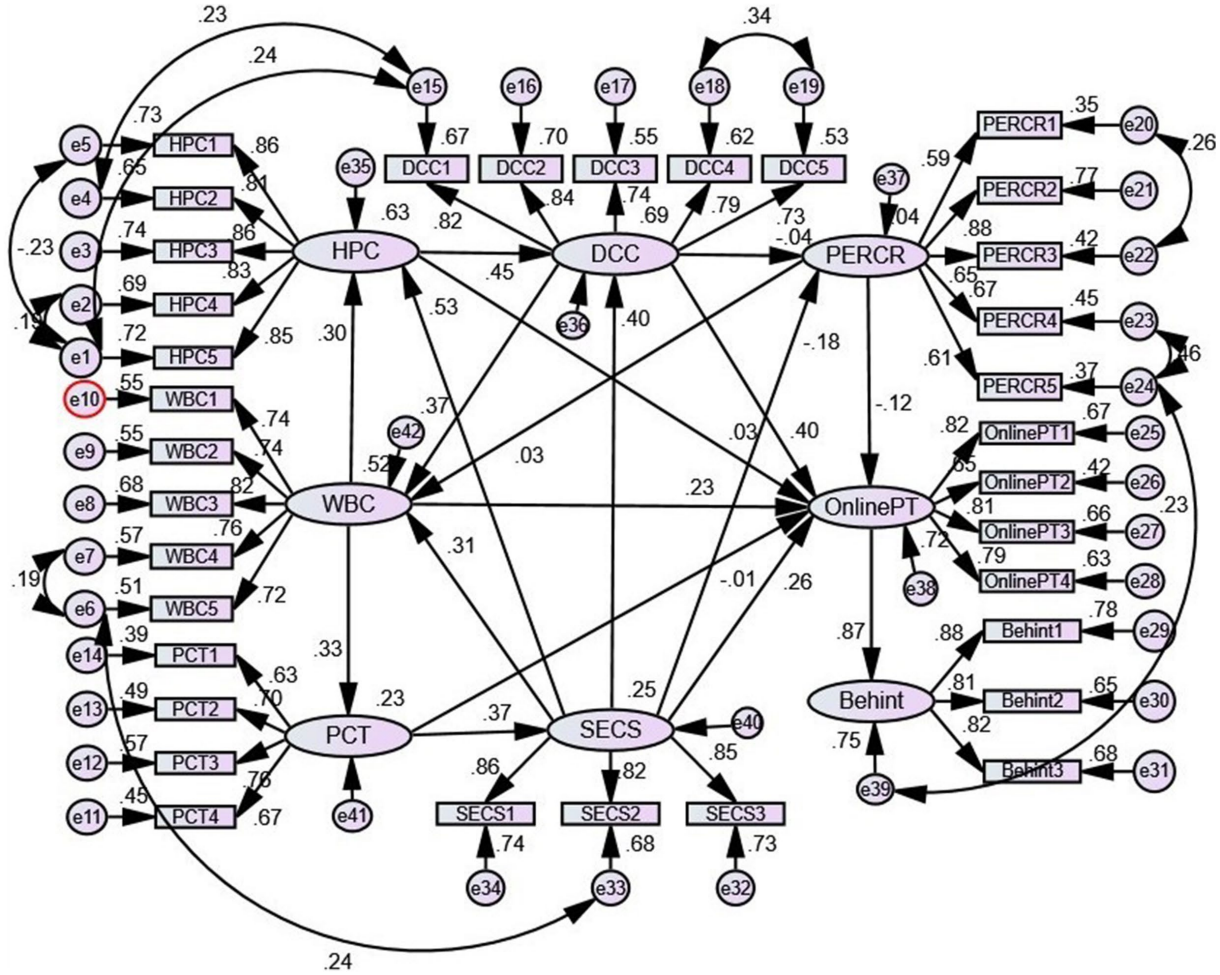
Structural equation model and standardized path coefficient.

The test result data in Figure 3 show path coefficients of online patients’ doctor trust, and website trust of 0.386 and 0.219, respectively, (*p* < 0.01), and hospital trust is 0.031 (*p* = 0.578). This shows that telemedicine, as the main body of medical services, has made great progress in improving medical quality, brand awareness, and medical environment. The path coefficient of the impact of institutional support on online patients is 0.257 (*p* < 0.01), which indicates that the institutional support and supervision mechanism of telemedicine will be gradually improved with the establishment and improvement of the social security system. The functions of the relevant state regulatory departments have lagged or the lack of service standards has improved, and online patients and the general public have achieved some results in warning government departments. Personal trust tendency has a positive impact on online patient trust, and the path coefficient for institutional guarantee is 0.392 (*p* < 0.01), indicating that the construction of institutional guarantee has been continuously strengthened and that appropriate publicity and education of relevant systems should be carried out to better promote online patients’ trust. Therefore, the impact of institutional safeguards on perceived risk and personal trust propensity of online patients needs to be accurately understood in practice.

### Consumer perceptions significantly increase online patient trust in telemedicine

The test results in Table 8 show that online patients have a high degree of trust in doctors, institutions, and websites, which shows that telemedicine, as the main body of medical services, has made great progress in improving medical quality, raising service awareness, promoting the progress of network technology, and improving the institution guarantee of the state. With the establishment and improvement of the social security system, the system guarantee and supervision mechanism for telemedicine will be gradually improved; the functions of the relevant state regulatory departments have lagged behind or service standards are missing, and online patients and the public have achieved certain results in warning government functional departments. Although personal trust tendency has no significant impact on online patient trust, it shows that system guarantee needs to be strengthened continuously, and promotion of and education in relevant systems should be carried out to better improve the perception characteristics of online patients and reduce the perception risks. Therefore, the impact of institutional safeguards on perceived risk and personal trust tendencies of online patients’ needs to be accurately grasped and strengthened in practice.

### Study’s limitations and future outlook

Despite extensive literature review and robust theoretical backing, this study exhibits shortcomings in the questionnaire survey and analysis. These deficiencies merit further refinement in subsequent research.

1. This study employed questionnaires via both online and offline channels. Differences in trust levels of mobile healthcare services may exist between respondents from online and offline surveys, yet this study did not delve into a comprehensive analysis. Future research could undertake a comparative analysis of the online trust dynamics between these two groups and investigate potential variations in their influencing factors.
2. This study aims to enhance the utilization of mobile healthcare services, satisfy patients’ healthcare needs, and mitigate the challenges of accessing and affording medical care. Mobile healthcare services are particularly beneficial for the senior citizens and those with chronic illnesses, yet this research did not specifically focus on or differentiate between these groups. Consequently, future studies can employ the latest health governance theories to categorize participants and undertake comprehensive research on specific demographics like the senior citizens, chronic patients, and expectant mothers, thereby developing targeted strategies to enhance trust based on the unique characteristics of these user groups.

## Conclusion and recommendations

Research findings indicate that the standardized factor loadings for majority of all dimensions presented in Table 4 are above 0.7 and exhibit significance; the composite reliability (C.R. values) exceed 0.7, and the average variance extraction (AVE values) are all greater than 0.5. In Table 6, the impact coefficients for doctor credibility, website credibility, hospital credibility, and institutional guarantee on online patient trust are 0.843, 0.692, 0.615, and 0.763, respectively, with *p* < 0.01, highlighting that trust in telemedicine services is crucial for online patient behavior choices. Perceived risk has a significant negative impact on online patients, and there is an urgent need to reduce the trust risk caused by environmental factors. In the development of telemedicine, hospital trust has no significant impact on online patients, and the public’s good image needs to be further improved. At the same time, personal trust propensity does not significantly increase online patient trust, and additional work is needed to establish sufficient public trust in telemedicine. Therefore, strengthening the construction of a multi-dimensional telemedicine trust system and enhancing the trust of online patients can effectively optimize the behavior intention of online telemedicine patients.

At present, telemedicine is an effective way to adjust the imbalance in the distribution of medical resources, speed up the construction of the grassroots medical and health system, and promote the equalization of medical services. It is an important part of the construction of health informatization. Because of their limited expertise and experience in telemedicine, online patients have a limited understanding of the costs, risks, and benefits of telemedicine services and related drugs. On the one hand, online patient trust mainly relies on subjective perceptions of one-sided evaluation to receive medical service information, and strengthens satisfaction with online medical care based on one’s own habitual, common-sense, and satisfaction cognition; on the other hand, online doctors and hospitals take advantage of their own medical service information and dominant power in pricing to carry out business regardless of the lack of relevant health knowledge of online patients under the circumstance of symmetric information, thus increasing the risk to online patients. Therefore, to protect the consumption of online patients, the government, medical institutions, and online patients should uphold the principle of good faith. In addition, online patients organically combine computer network communication technology with modern medicine through telemedicine to form an efficient medical service method, which plays a positive role in alleviating uneven distribution of medical resources, insufficient services in medical institutions, and medical expenses. Mitigation effects, for example, are like public health security crises such as the COVID-19 pandemic. Moreover, telemedicine has epoch-making value and significance for long-term public health governance and residents’ health benefits.

In summary, the TPB and TAM theory models are distinct frameworks with different focal points. In practical applications, we must choose the most suitable model based on research questions and data conditions to interpret and predict online patient trust. Additionally, we must be mindful of the model’s assumptions and limitations, avoiding overinterpretation and overapplication. As behavioral science and information technology continue to evolve, we anticipate the emergence and development of additional telemedicine theory models to more effectively explain and predict human online medical behavior and information technology acceptance.

## Data Availability

The original contributions presented in the study are included in the article/supplementary material, further inquiries can be directed to the corresponding author.
